# Prognostic Value of Systemic Inflammatory Response Markers for CIN2+ Recurrence After Loop Electrosurgical Excision Procedure: A Retrospective Cohort Study

**DOI:** 10.3390/jcm14124059

**Published:** 2025-06-08

**Authors:** Sevim Ezgi Katran, Kevser Arkan, Süleyman Cemil Oğlak, İpek Betül Özçivit Erkan, Gözde Cebeci, Engin Çelik

**Affiliations:** 1 Department of Obstetrics and Gynecology, Health Sciences University, Kanuni Sultan Suleiman Training and Research Hospital, Istanbul 34303, Turkey; gozde.cebeci@saglik.gov.tr; 2 Department of Gynecologic Oncology, Health Sciences University, Gazi Yaşargil Training and Research Hospital, Diyarbakır 21500, Turkey; kevser.toprak1989@gmail.com; 3 Department of Obstetrics and Gynecology, Health Sciences University, Gazi Yaşargil Training and Research Hospital, Diyarbakır 21500, Turkey; drcemiloglak@gmail.com; 4 Department of Obstetrics and Gynecology, Kızıltepe State Hospital, Mardin 47400, Turkey; ipekbetulozcivit@gmail.com; 5 Department of Gynecologic Oncology, Health Sciences University, Kanuni Sultan Suleiman Training and Research Hospital, Istanbul 34303, Turkey; ecelik81@istanbul.edu.tr

**Keywords:** loop electrosurgical excision procedure, systemic inflammatory response parameters, cervical intraepithelial neoplasia, recurrence

## Abstract

**Objectives:** To evaluate the prognostic value of systemic inflammatory response (SIR) parameters in predicting the recurrence of cervical intraepithelial neoplasia (CIN2+) in women undergoing a loop electrosurgical excision procedure (LEEP). **Methods:** This retrospective study included women aged ≥18 years who underwent an LEEP at a tertiary center between 2013 and 2023. Patients who were pregnant and those who had malignancies, immune disorders, or prior cervical surgery were excluded. The data collected included age, parity, cervical cytology, HPV DNA status, histology, LEEP specimen size, and preoperative blood count parameters. Follow-up was performed every six months using cytology, colposcopy, and histology to assess recurrence. The SIR markers evaluated included the neutrophil-to-lymphocyte ratio (NLR), lymphocyte-to-monocyte ratio (LMR), platelet-to-lymphocyte ratio (PLR), and lymphocyte count. Statistical analyses included ROC curves and Cox regression. **Results:** Of the 1068 patients included, 726 had follow-up data, and 32 (4.4%) experienced a recurrence after a mean interval of 24 ± 20 months. Recurrence-negative patients had higher median lymphocyte counts (2.40 vs. 2.15, *p* = 0.031) and LMRs (4.57 vs. 3.86, *p* = 0.011). The disease-free survival period was longer in patients with high lymphocyte counts, a low NLR and PLR, and a high LMR. However, the discriminatory power of these markers was limited. In the multivariate analysis, only a PLR > 118.4 remained independently associated with an increased recurrence risk (HR 3.06, *p* = 0.011). Due to the small number of cases of recurrences and the small amount of HPV DNA results, the findings should be interpreted with caution. **Conclusions:** Preoperative SIR markers such as the PLR, NLR, LMR, and lymphocyte count showed statistical associations with CIN2+ recurrence after an LEEP, but their clinical utility appears to be limited. Further prospective studies are needed to validate these findings.

## 1. Introduction

Cervical intraepithelial neoplasia (CIN) is a precancerous lesion of the cervix, diagnosed through cervical biopsy. It has been shown that 99.7% of malignancies are associated with human papillomavirus (HPV) [1,2]. Low-grade CIN1 has a low potential for progression to malignancy and is often associated with spontaneous regression, whereas CIN2 and CIN3 have a higher risk of progression to malignancy, with rates of 5% and 12%, respectively [3,4,5]. Recent evidence suggests that CIN1 does not independently increase the risk of progression to CIN3 beyond that conferred by the underlying HPV genotype [6], and that the risk of developing CIN2+ during follow-up is similar in women with CIN1 and those with a negative biopsy, supporting conservative management [7].

Cervical cancer can be prevented with early diagnosis and treatment by screening for preinvasive lesions [8,9]. The loop electrosurgical excision procedure (LEEP) is the most widely used excisional treatment method for diagnosing and treating cervical precancerous lesions. It enables the removal of the lesion using a thin, ring-shaped wire electrocautery, followed by diagnosis through histopathologic examination [10]. Meta-analyses report that the recurrence rate of CIN after an LEEP is approximately 26.6% at the 12-month follow-up and 5.3% over a long-term follow-up period. Therefore, given the risk of high-grade cervical neoplasms progressing to malignancy, patient follow-up after an LEEP is crucial [11,12].

The complex interplay between inflammation and cancer plays a pivotal role in tumor development and progression. The tumor microenvironment produces proinflammatory cytokines that attract macrophages, neutrophils, T and B lymphocytes, and natural killer (NK) cells [13,14,15,16]. Leukocytosis and neutrophilia are the most common systemic changes that are associated with disease prognosis. Around 20% of all cancers are linked to chronic inflammatory processes triggered by bacterial and viral infections or environmental factors such as tobacco use or autoimmune diseases [17]. Systemic inflammatory response (SIR) parameters, including serum markers such as the neutrophil-to-lymphocyte ratio (NLR), lymphocyte-to-monocyte ratio (LMR), platelet-to-lymphocyte ratio (PLR), and lymphocyte count, have been shown to serve as prognostic markers in many malignancies [18]. Increased NLR and PLR contribute to cancer development by stimulating vascularization and tumor cell proliferation in patients with preinvasive cervical pathologies [19]. Low levels of lymphocytes are associated with cancer progression, as lymphocytes inhibit tumor growth by promoting cytotoxic T-cell activity [20]. However, SIR markers may be influenced by broader patient-related variables such as obesity, smoking, physical inactivity, and psychosocial stress, which are often underrecognized in clinical risk models—highlighting the need to interpret their prognostic value within a more holistic, patient-centered context [21].

These results suggest that SIR markers may serve as potential indicators for different stages of precancerous cervical lesions and could play a significant role in predicting prognoses [18,22]. While many parameters have been investigated to predict disease progression in cervical cancer, no large studies have specifically examined the factors affecting prognosis in terms of CIN in patients undergoing an LEEP. In our study, we aimed to assess the impact of SIR markers on the recurrence and prognosis of CIN in patients undergoing an LEEP.

## 2. Materials and Methods

This retrospective study included women who presented to the gynecology outpatient clinic at Health Sciences University, Kanuni Sultan Süleyman Training and Research Hospital between November 2013 and May 2023, meeting the following criteria: women over 18 years of age who had undergone LEEP. Exclusion criteria included those with cervical lesions suspicious in terms of malignancy; pregnancy; a history of malignancy, immune system diseases, or other sexually transmitted infections; and having undergone a hysterectomy, cervical surgery, or chemotherapy. The medical records of the included patients were reviewed retrospectively. The following data were recorded from the medical database: age, parity, cervical cytology results, HPV DNA results, histological colposcopy + endocervical curettage (ECC) findings, LEEP specimen size, LEEP + ECC results (surgical margins and gland involvement), and preoperative complete blood count parameters, including hemoglobin, hematocrit, red blood cell count, white blood cell count, platelets, neutrophils, lymphocytes, cervical cytology results post-LEEP, HPV DNA results post-LEEP, histological colposcopy + ECC findings post-LEEP, follow-up LEEP + ECC results, and follow-up duration.

LMR was defined as the lymphocyte count divided by the monocyte count. NLR was defined as the neutrophil count divided by the lymphocyte count. PLR was defined as the platelet count divided by the lymphocyte count. For patients with positive surgical margins after LEEP, re-LEEP or hysterectomy was planned. If abnormalities were detected in follow-up cervical cytology or HPV DNA results, colposcopic biopsy and ECC were performed. Recurrence of CIN was defined as the presence of CIN2 or CIN3 lesions in follow-up colposcopic biopsies post-LEEP. For patients without recurrence, the end of follow-up was determined as the date of the last negative cytology. Patients were followed every six months with pelvic and ultrasound examinations, cervical cytology, colposcopy, and additional LEEP procedures, if necessary, at the gynecologic oncology department. Cytology was performed using liquid-based cytology and analyzed by the pathology department of our hospital. Results were reported according to the Bethesda system [23]. Patients with positive HPV DNA results for types 16 and 18, as well as any other HPV DNA positivity, along with cervical cytology results showing atypical squamous cells of undetermined significance (ASCUS) or higher, were referred for colposcopic examination. Biopsy specimens from colposcopy were analyzed by the pathology department and reported using the Bethesda System 2001 terminology [23]. Recurrent disease was defined as CIN2 or higher lesions detected 12 months after LEEP, while residual disease was defined as CIN1 after LEEP or CIN2 or higher lesions detected within 12 months following LEEP [18]. The 2019 ASCCP Risk-Based Management Consensus Guidelines for Abnormal Cervical Cancer Screening Tests and Cancer Precursors was followed when determining post-LEEP follow-up and decisions regarding repeat excision or hysterectomy [24].

All procedures involving human participants were conducted in accordance with the 1964 Helsinki Declaration and its later amendments or with comparable ethical standards. Written informed consent was obtained from all participants. Ethical approval was granted by the Health Sciences University, Kanuni Sultan Suleiman Training and Research Hospital Ethics Committee (approval number: KAEK/2023.07.80, date: 12 July 2023).

### 2.1. Sample Size

The sample size calculation was based on the results of a previous study that investigated preoperative PLR levels for predicting the recurrence of HSIL after LEEP [16]. Using G-Power 3.1 and assuming an effect size of 1.0807, a significance level (α) of 0.05, and a power of 0.95, the required sample size was estimated to be 566.

### 2.2. Statistical Analysis

The statistical analysis was conducted using the R program (version 4.2.0) (R: A Language and Environment for Statistical Computing, R Foundation for Statistical Computing, Vienna, Austria, available online at http://www.r-project.org/). The following packages were utilized for the analysis: gtsummary v1.6.0, survival v3.5.5, survminer v0.4.9, and pROC v1.18.0 [25,26,27,28]. The normality of the distribution of the parameters was assessed using the Kolmogorov–Smirnov and Shapiro–Wilk tests. Continuous variables were presented as median (25th and 75th quartiles) and mean ± standard deviation. Categorical variables were presented as frequency (percentage). Continuous variables were compared using the Mann–Whitney U test, while categorical variables were compared using the Pearson chi-square test or Fisher’s exact test, as appropriate. In cases in which processor capacity was insufficient, *p*-values were simulated.

The discriminative capacity of the parameters for the presence of recurrence was determined using ROC curve analysis. The area under the curve (AUC) values were interpreted as follows: <0.6 indicated no discrimination, 0.6–0.7 indicated poor discrimination, 0.7–0.8 indicated acceptable discrimination, 0.8–0.9 indicated excellent discrimination, and 0.9–1 indicated outstanding discrimination [29]. The “surv_cutpoint” function was used for survival analysis based on determined cut-off values of certain parameters [27]. Log-rank Kaplan–Meier analysis was performed to compare disease-free survival (DFS) and cumulative incidence rates during the follow-up period. Factors affecting the risk of recurrence were evaluated using univariate Cox regression analysis. Multiple Cox regression analysis was conducted with variables that were significant in the univariate analysis. A *p*-value of <0.05 was considered significant.

## 3. Results

A total of 1068 patients who underwent surgery as part of an LEEP were included in the study. The mean age of the patients was 41 ± 10 years. The demographic and preoperative findings are presented in Table 1. Preoperative HPV-DNA testing revealed positivity for types 16 and/or 18 in 364 patients (72%), while postoperatively, HPV-DNA positivity for types 16 and/or 18 was found in 14 patients (25%). The histology results from the cervical biopsies showed that CIN2 was the most common diagnosis, found in 518 patients (50%), followed by CIN3 in 237 patients (23%). The endocervical curettage (ECC) results were predominantly negative, reported in 858 patients (84%).

The mean LEEP specimen size was 0.95 ± 0.43 cm, with a median of 1.00 cm (0.70–1.00). The final histopathology results from the LEEP specimens showed CIN3 in 299 patients (28%), CIN2 in 286 patients (27%), CIN1 in 225 patients (21%), and no dysplasia in 258 patients (24%). Surgical margins were negative in 980 patients (91.8%), and endocervical gland involvement was absent in 836 patients (78%).

### 3.1. Comparison of Recurrent and Non-Recurrent Cases

A total of 342 patients (32%) were lost to follow-up after the LEEP. Among the 726 patients who continued follow-up, only 32 (4.4%) experienced a recurrence. The mean duration between the LEEP operation and recurrence was 24 ± 20 months, with a median of 18 months (9–32). Among the patients who experienced a recurrence, 23 (72%) had CIN2, and 9 (28%) had CIN3 based on the recurrent LEEP pathology results.

Among the patients who were followed up with, those with recurrences were compared to those without recurrences in terms of demographic and cytological results, along with total blood count parameters (Table 2). There was no statistically significant difference in the median age between the two groups; the recurrence-positive group had a median age of 42 years (36–44), while the recurrence-negative group had a median age of 39 years (34–45) (*p* = 0.732). Regarding total blood count parameters, the recurrence-negative group had a higher median lymphocyte value compared to the recurrence-positive group (2.40 [1.90–2.80] vs. 2.15 [1.60–2.42], *p* = 0.031). Additionally, the lymphocyte–monocyte ratio (LMR) was higher in the recurrence-negative group (4.57 [3.72–5.62] vs. 3.86 [3.18–5.00], *p* = 0.011).

**Figure 1:**
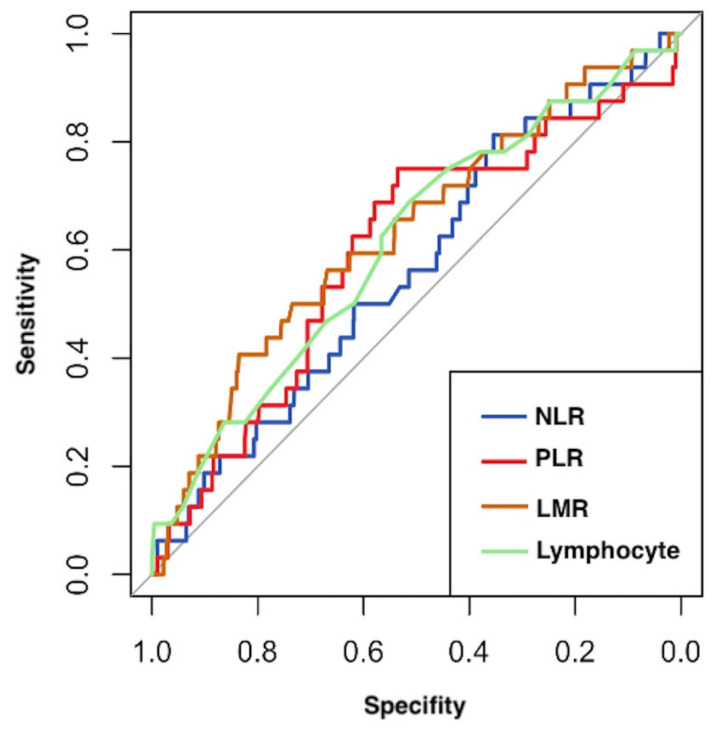
ROC curves for the neutrophil-to-lymphocyte ratio (NLR), platelet-to-lymphocyte ratio (PLR), lymphocyte-to-monocyte ratio (LMR), and lymphocyte count assessing the discriminatory power of these parameters in predicting recurrence in patients.

In Figure 1, the ROC curves for the lymphocyte count, NLR, PLR, and LMR are presented. The AUC for the lymphocyte count was 0.613 (95% CI: 0.51–0.716), indicating poor discrimination. The AUC for the NLR was 0.565 (95% CI: 0.465–0.665), which is considered nondiscriminatory. The AUC for the PLR was 0.600 (95% CI: 0.493–0.706), while the AUC for the LMR was 0.632 (95% CI: 0.529–0.736), both suggesting poor discrimination.

### 3.2. Comparison of DFS Based on SIR Parameters

**Figure 2:**
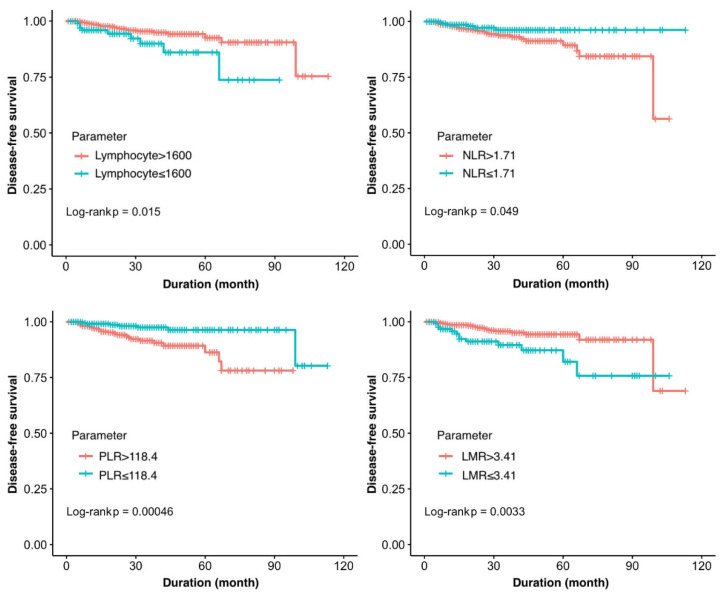
Comparison of disease-free survival (DFS) based on lymphocyte count, neutrophil-to-lymphocyte ratio (NLR), platelet-to-lymphocyte ratio (PLR), and lymphocyte-to-monocyte ratio (LMR).

In Figure 2, a comparison of DFS based on lymphocyte count, NLR, PLR, and LMR values is presented using a Kaplan–Meier analysis. The mean DFS was 94.9 ± 6.7 months in patients with lymphocyte counts ≤ 1600 cells/µL, compared to 103.8 ± 2.5 months in those with counts > 1600 cells/µL (*p* = 0.015). The mean DFS for women with a preoperative NLR ≤ 1.71 was 109.4 ± 1.5 months, while it was 98.0 ± 3.9 months for those with a preoperative NLR > 1.71 (*p* = 0.049). In patients with a preoperative PLR ≤ 118.4, the mean DFS was 107.6 ± 2.4 months, whereas it was 97.9 ± 3.5 months for women with a PLR > 118.4 (*p* < 0.001). The DFS in women with a preoperative LMR ≤ 3.41 was 95.4 ± 5 months, compared to 103.7 ± 3.2 months for those with a preoperative LMR > 3.41 (*p* = 0.003).

On the other hand, the comparisons made between groups according to age (>30, 30–50, and >50 years), gland involvement, surgical margin positivity, LEEP results, the size of the LEEP specimen, preoperative HPV DNA, and postoperative HPV DNA positivity for types 16 and/or 18, as well as recurrence pathology results, yielded *p*-values of 0.745, 0.641, 0.673, 0.15, 0.135, 0.409, 0.304, and 0.328, respectively. However, when comparing the postoperative HPV DNA 16 and/or 18 positive group with the HPV DNA 16/18 negative group in terms of DFS, a statistically significant difference was observed (*p* = 0.0094).

### 3.3. Evaluation of Factors Associated with Recurrence

Factors affecting recurrence were evaluated using univariate and multivariate Cox regression analyses (Table 3). According to the univariate Cox regression analysis, lymphocyte counts < 1600 cells/µL, an NLR > 1.71, a PLR > 118.4, and an LMR ≤ 3.41 were statistically significant factors affecting the recurrence rate, with the following hazard ratios (HRs): lymphocyte < 1600 cells/µL (HR 2.53, *p* = 0.030), an NLR > 1.71 (HR 2.38, *p* = 0.038), a PLR > 118.4 (HR 4.02, *p* < 0.001), and an LMR ≤ 3.41 (HR 2.77, *p* = 0.007). However, in the multivariate regression analysis, only a PLR > 118.4 was found to be associated with an increased recurrence rate, with an HR of 3.06 (*p* = 0.011).

## 4. Discussion

We investigated the recurrence of CIN2+ following an LEEP and its association with SIR parameters. High preoperative lymphocyte counts and a high LMR were associated with a longer time to recurrence, while a low NLR and PLR were linked to improved disease-free survival. However, the discriminatory power of these markers was limited. Prior studies have demonstrated associations between SIR markers and prognosis in endometrial and cervical cancers [18,19,30,31]. Our findings suggest that certain preoperative SIR markers may have prognostic value for recurrence after an LEEP, although their clinical utility remains limited.

Several studies have examined the relationship between SIR markers, HPV infection, and the recurrence of CIN following an LEEP. Peripheral lymphocyte activity has been suggested to reflect the host immune response to viral infections. In a 2023 study, Huang et al. investigated 381 patients with CIN2–3 treated with an LEEP and found that recurrence (defined as CIN2–3 reappearance after 12 months or residual CIN1–3 within 12 months) was more common in women with persistent high-risk HPV infection and an elevated PLR. After five years, persistent hrHPV infection increased the risk of recurrence by 3.9 fold, and an elevated PLR was associated with a 2.1-fold increased risk [18]. Similarly, Chun et al. reported that in 230 women with CIN1–3 undergoing an LEEP, the disease-free survival was significantly lower in those with a high preoperative NLR compared to those with a low NLR [31]. Farzaneh et al., in a cohort of 317 patients, found that an elevated NLR and total white blood cell count were significantly associated with reduced disease-free survival; however, in contrast to our findings, the PLR was not significantly associated with outcomes in their study [32]. While our results are broadly consistent with these previous studies, our cohort did not include patients with CIN1 residual disease and our results were based on a larger sample size. Overall, the literature provides growing evidence that SIR markers may serve as independent prognostic indicators of disease-free survival after surgical treatment for CIN.

The relationship between SIR markers and invasive cervical cancer has also been explored. In one study involving 110 patients with LSIL, HSIL, or invasive cervical cancer, PLRs were significantly higher in patients with invasive cancer compared to those with preinvasive lesions or normal cytology [19]. Both an elevated NLR and PLR were found to be predictive of cervical cancer. Although that study did not evaluate the LMR or lymphocyte count and had a limited sample size, it was among the first to suggest a role for the PLR and NLR in distinguishing preinvasive from invasive cervical disease. Based on our findings and previous studies, we propose that hemogram-derived inflammatory markers such as the NLR, PLR, LMR, and lymphocyte count may serve as inexpensive, accessible, and potentially useful prognostic tools for assessing the risk of CIN recurrence after excisional treatment. However, their discriminatory value in clinical practice appears to be limited, and further validation in larger prospective cohorts is needed.

Persistent HPV infection has been identified by other studies as an important predictor of CIN prognosis. Our findings also confirm that after an LEEP, patients who experienced a recurrence had postoperative positivity for HPV types 16 and 18, which correlated with a lower disease-free survival period. Specifically, infections with HPV types 16, 18, 31, 33, 56, and 58 may significantly contribute to the progression and recurrence of HSIL [33,34,35]. Furthermore, persistent HPV disease was associated with an elevated SIR, NLR, PLR, and other hematologic parameters [36].

The technique used for cervical conization significantly affects margin status and recurrence risk due to differences in thermal damage and excision precision. Studies consistently show that CO_2_ laser conization is associated with lower rates of positive endocervical or deep margins compared to cold knife (CK) and the LEEP, particularly in squamous lesions [37]. While some evidence suggests no clear association between surgical method and margin status [38], a recent meta-analysis indicated that CK and laser conization had lower treatment failure rates compared to an LEEP [39]. These findings suggest that while clinical outcomes like recurrence may be comparable, the laser and cold knife methods may offer superior efficacy in preventing treatment failure. However, it should be kept in mind that World Health Organization (WHO) recommends the LEEP over cryotherapy because the LEEP not only treats CIN effectively but also provides a tissue specimen for histopathological examination, allowing for a more accurate diagnosis and follow-up—an advantage that cryotherapy does not offer [40].

This study is limited by its retrospective design and single-center setting. Although patients with known malignancies, ongoing chemotherapy, immune system disorders, other sexually transmitted infections, and immunosuppression were excluded to reduce potential confounding variables, several important factors influencing systemic inflammation, such as smoking status, were not controlled for. The SIR may also be affected by non-cancer-related factors, including comorbid conditions (obesity or concurrent infections) and lifestyle factors, which were not thoroughly considered in this analysis. While the sample size is relatively large and the statistical power is adequate, these strengths are offset by the substantial loss to follow-up and the very low incidence rate of recurrence, which limit the robustness of the findings.

In particular, the predictive value of the SIR markers appears to be modest, with limited discriminatory ability, and this should be acknowledged more explicitly. Furthermore, the amount of available HPV DNA results, especially postoperatively, was too small to support meaningful conclusions and should be omitted from the main analysis. Although the follow-up duration of two years is relatively long compared to that of similar studies, the clinical utility of the evaluated SIR parameters remains uncertain and requires further validation in prospective cohorts.

The preoperative lymphocyte count, LMR, and PLR showed statistically significant associations with recurrence risk and disease-free survival following an LEEP. However, the clinical relevance of these findings appears to be limited due to the low incidence rate of recurrence and the modest discriminatory power of the markers. While SIR parameters are inexpensive and readily available as part of routine blood tests, their role as reliable prognostic tools in clinical practice remains uncertain. Future research should focus on validating these findings in larger, prospective cohorts with standardized follow-up and on integrating inflammatory markers with established risk factors, such as HPV persistence, to improve individualized follow-up and management strategies. A growing trend in the management of early-stage cervical cancer emphasizes reducing surgical morbidity while maintaining oncologic safety [19]. In this context, our findings on the predictors of recurrence risk may contribute to more personalized and less invasive treatment strategies.

## 5. Conclusions

In conclusion, certain preoperative SIR markers, such as lymphocyte count, LMR, NLR, and PLR, showed statistically significant associations with CIN2+ recurrence after an LEEP. However, their discriminatory power was limited, and the clinical utility of these markers remains uncertain. Hematologic markers are simple and inexpensive to obtain, but further prospective, multicenter studies with standardized follow-up are needed to determine whether they can reliably inform individualized risk assessments and follow-up strategies in patients treated for high-grade cervical lesions.

## Figures and Tables

**Table 1:** Preoperative cervical cytology, HPV DNA status, histology from cervical biopsies and ECC, and LEEP histopathology in the total cohort.

Variables		n (%)
Preoperative cervical cytology	NILM	303 (29.0%)
	Low-grade cytology (ASCUS/LSIL)	346 (33.0%)
	High-grade cytology (ASC-H/HSIL/AGC)	370 (35.3%)
	Insufficient	17 (1.6%)
Preoperative HPV-DNA *	HPV-DNA 16 and/or 18 positive	364 (72)
	Other HPV-DNA types positive	134 (27)
	Negative	5 (1.0)
Postoperative HPV-DNA **	HPV-DNA 16 and/or 18 positive	14 (25)
	Other HPV-DNA types positive	5 (8.9)
	Negative	37 (66)
Histological results of colposcopy	CIN1	138 (13)
	CIN2	518 (50)
	CIN3	237 (23)
	Negative	133 (13)
Histological results of colposcopy + ECC	CIN1	43 (4.2)
	CIN2	72 (7.0)
	CIN3	53 (5.2)
	Negative	858 (84)
LEEP	CIN1	225 (21)
	CIN2	286 (27)
	CIN3	299 (28)
	Negative	258 (24)
LEEP + ECC	CIN1	19 (1.8)
	CIN2	20 (1.9)
	CIN3	18 (1.7)
	Negative	1011 (95)
Surgical margin	Negative	980 (91.8)
	Positive	88 (8.2)
Gland involvement	Negative	836 (78)
	Positive	232 (22)

* A total of 565 of the 1068 patients (52.9%) did not have preoperative HPV-DNA results. ** 1012 patients of the 1068 patients (94.8%) did not have postoperative HPV-DNA results. AGC: atypical glandular cell, AGC-NOS atypical glandular cell (not otherwise specified), ASC-H: atypical squamous cell (cannot exclude high-grade squamous intraepithelial lesion), ASCUS: atypical squamous cell of undetermined significance, CIN: cervical intraepithelial neoplasia, ECC: endocervical curettage, HPV-DNA: human papilomavirus deoxyribonucleic acid, HSIL: high-grade squamous intraepithelia lesion, LEEP: loop electrosurgical excision procedure, LSIL: low-grade squamous intraepithelia lesion, https://www.healthline.com/health/low-grade-squamous-intraepithelial-lesion (accessed on 25 April 2025), NILM: negative for intraepithelial lesion or malignancy.

**Table 2:** Comparison of demographic characteristics, cervical cytology, histology from cervical biopsies and ECC, LEEP histopathology, and total blood count parameters between patients with and without recurrent CIN2+ following LEEP.

Variables		Recurrence-Positive (N = 32) n (%)	Recurrence-Negative (N = 694) n (%)	*p*
Age	<30	3 (9.4)	59 (8.5)	0.548 ^a^
	30–50	27 (84)	543 (78)	
	>50	2 (6.2)	92 (13)	
Preoperative cervical cytology	NILM (Negative)	8 (25.0%)	183 (26.4%)	0.801 ^b^
	Low-grade cytology (ASCUS/LSIL)	13 (40.6%)	244 (35.2%)	
	High-grade cytology (ASC-H/HSIL/AGC)	10 (31.3%)	236 (34.0%)	
	Insufficient	0 (0.0%)	14 (2.0%)	
Preoperative HPV-DNA	HPV-DNA 16 and/or 18 positive	13 (76)	240 (73)	1.0 ^a^
	Other HPV-DNA types positive	4 (24)	86 (26)	
	Negative	0 (0)	3 (0.9)	
Postoperative HPV-DNA	HPV-DNA 16 and/or 18 positive	1 (100)	13 (24)	0.339 ^a^
	Other HPV-DNA types positive	0 (0)	5 (9.1)	
	Negative	0 (0)	37 (67)	
Histological results of colposcopy	CIN1	3 (11)	100 (15)	0.650 ^a^
	CIN2	16 (57)	331 (49)	
	CIN3	8 (29)	166 (25)	
	Negative	1 (3.6)	72 (11)	
Histological result of colposcopy + ECC	CIN1	1 (3.6)	30 (4.5)	0.050 ^a^
	CIN2	6 (21)	44 (6.6)	
	CIN3	1 (3.6)	35 (5.2)	
	Negative	20 (71)	559 (84)	
LEEP	CIN1	3 (9.4)	148 (21)	0.134 ^a^
	CIN2	12 (38)	193 (28)	
	CIN3	13 (41)	204 (29)	
	Negative	4 (12)	149 (21)	
LEEP + ECC	CIN1	1 (3.1)	13 (1.9)	0.320 ^b^
	CIN2	1 (3.1)	10 (1.4)	
	CIN3	1 (3.1)	12 (1.7)	
	Negative	29 (91)	659 (95)	
Surgical margin	Negative	30 (94)	638 (92)	1.0 ^b^
	Positive	2 (6.2)	56 (8.1)	
Gland involvement	Negative	24 (75)	538 (78)	0.739 ^c^
	Positive	8 (25)	156 (22)	
		**Median (25th–75th quartile)**	**Median (25th–75th quartile)**	
LEEP specimen size (cm)		0.90 (0.58–1.13)	1.00 (0.70–1.00)	0.840 ^d^
Follow-up duration (month)		18 (9–31)	22 (10–41)	0.270 ^d^
Hemoglobin (g/dL)		12.90 (12.20–13.72)	12.90 (12.10–13.60)	0.869 ^d^
Hematocrit (%)		39.0 (36.9–42.1)	38.7 (36.5–40.7)	0.456 ^d^
RBC (10^6^/µL)		4.64 (4.40–4.90)	4.55 (4.30–4.80)	0.161 ^d^
MCV (fL)		85 (81–87)	85 (82–89)	0.331 ^d^
Platelet (10^3^/µL)		261 (227–306)	273 (234–315)	0.385 ^d^
WBC (10^3^/µL)		7.51 (6.08–8.33)	7.71 (6.37–9.27)	0.193 ^d^
Neutrophil (10^3^/µL)		4.38 (3.50–5.16)	4.52 (3.57–5.70)	0.438 ^d^
Monocyte (10^3^/µL)		0.54 (0.44–0.61)	0.50 (0.40–0.64)	0.746 ^d^
Eosinophil (10^3^/µL)		0.12 (0.08–0.20)	0.12 (0.08–0.21)	0.886 ^d^
Lymphocyte (10^3^/µL)		2.15 (1.60–2.42)	2.40 (1.90–2.80)	**0.031 ^d^**
NLR		2.07 (1.74–2.68)	1.94 (1.53–2.50)	0.213 ^d^
PLR		136 (113–155)	116 (95–145)	0.057 ^d^
LMR		3.86 (3.18–5.00)	4.57 (3.72–5.62)	**0.011 ^d^**

^a^ Fisher’s exact test, ^b^ Fisher’s exact test (simulated by 10,000 iterations), ^c^ Pearson’s chi-squared test, ^d^ Mann–Whitney U test. AGC: atypical glandular cell, ASC-H: atypical squamous cell—cannot exclude high-grade squamous intraepithelial lesion, ASCUS: atypical squamous cell of undetermined significance, CIN: cervical intraepithelial neoplasia, ECC: endocervical curettage, HPV-DNA: human papillomavirus deoxyribonucleic acid, HSIL: high-grade squamous intraepithelial lesion, LEEP: loop electrosurgical excision procedure, LMR: lymphocyte–monocyte ratio, LSIL: low-grade squamous intraepithelial lesion, MCV: mean corpuscular volume, NILM: negative for intraepithelial lesion or malignancy, NLR: neutrophil-to-lymphocyte ratio, PLR: platelet-to-lymphocyte ratio, RBC: red blood cell, WBC: white blood cell.

**Table 3:** Univariate and multivariate Cox regression analysis for recurrence.

Variables		Univariate		Multivariate	
		HR (%95 CI)	*p*	HR (%95 CI)	*p*
**Age**	**<30**		0.718		
	**30–50**	0.87 (0.26–2.88)			
	**>50**	0.52 (0.09–3.12)			
**Preoperative HPV-DNA**	**Negative**		0.845		
	**HPV-DNA 16 and/or 18 positive**	0.81 (0.06–11.1)			
	**Other HPV-DNA types positive**	0.60 (0.05–8.06)			
**Postoperative HPV-DNA**	**Negative**		0.424		
	**HPV-DNA 16 and/or 18 positive**	1.37 (0.08–22.8)			
	**Other HPV-DNA types positive**	8.21 (0.38–176)			
**LEEP**	**Negative**		0.123		
	**CIN1**	0.82 (0.18–3.67)			
	**CIN2**	2.36 (0.76–7.33)			
	**CIN3**	2.36 (0.77–7.24)			
**Surgical margin positivity**		0.74 (0.18–3.08)	0.660		
**Gland involvement**		1.21 (0.54–2.70)	0.646		
**Lymphocyte ≤ 1600 cell/µL)**		2.53 (1.16–5.50)	0.030 *	1.21 (0.52–2.82)	0.655
**NLR > 1.71**		2.38 (0.98–5.79)	0.038 *	1.41 (0.55–3.63)	0.467
**PLR > 118.4**		4.02 (1.73–9.35)	<0.001 *	3.06 (1.23–7.58)	0.011 *
**LMR ≤ 3.41**		2.77 (1.36–5.63)	0.007 *	1.84 (0.85–3.95)	0.127
**LEEP specimen size >1.4 cm**		1.90 (0.81–4.47)	0.166		

CI: confidence interval, HPV-DNA: human papilomavirus deoxyribonucleic acid, HR: hazard ratio, LEEP: loop electrosurgical excision procedure, LMR: lymphocyte–monocyte ratio, NLR: neutrophile-to-lymphocyte ratio, PLR: platelet-to-lymphocyte ratio. * Statistically significant (*p* < 0.05).

## Data Availability

The data that support the findings of this study are available from the corresponding author (S.E.K.) upon reasonable request.
